# The Impact of Surgery Delay on Early-Stage Ovarian Cancer

**DOI:** 10.3390/life15010122

**Published:** 2025-01-17

**Authors:** Dimitrios Zouzoulas, Dimitrios Tsolakidis, Iliana Sofianou, Tilemachos Karalis, Michalis Aristotelidis, Panagiotis Tzitzis, Evangelia Deligeoroglou, Maria Topalidou, Eleni Timotheadou, Grigoris Grimbizis

**Affiliations:** 1 1st Department of Obstetrics & Gynecology, Aristotle University of Thessaloniki, “Papageorgiou” Hospital, 564 29 Thessaloniki, Greece; 2 Radiotherapy Department, “Papageorgiou” Hospital, 564 29 Thessaloniki, Greece; 3 Department of Oncology, Aristotle University of Thessaloniki, “Papageorgiou” Hospital, 564 29 Thessaloniki, Greece

**Keywords:** surgery delay, early-stage ovarian cancer, diagnosis, adnexal mass

## Abstract

(1) Background: Suspicious adnexal masses should be referred to gynecological oncology units. However, when surgery waiting lists are prolonged, these patients usually suffer from a delay in surgery. This could have a negative impact on their prognosis when the final diagnosis is ovarian cancer (OC). The primary aim of this study was to investigate the impact of surgery delay on the oncological results of early-stage ovarian cancer patients. (2) Methods: We retrospectively reviewed the records of early-stage OC patients who underwent surgery in the 1st Department of Obstetrics and Gynecology from 2012 to 2019. Time to surgery was defined as the time interval from the day of first examination to the day of surgery. (3) Results: A total of 72 patients were categorized into two groups, with a cut-off point of 5 weeks: 32 were treated ≤ 5 weeks (group A), and 40 > 5 weeks (group B). Concerning age, BMI or comorbidities, no differences were found between the two groups. Furthermore, no differences were presented in the post-operative complications rate, hospital stay, ICU admittance, or in disease-free (*p* = 0.48) and overall survival rates (*p* = 0.703). (4) Conclusions: Suspicious adnexal masses should undergo careful differential diagnosis to avoid delays in the “wait and see” period when the final diagnosis is positive for malignancy. However, the time to surgery for early-stage OC over 5 weeks seems to be relatively safe, with no impact on the mortality, morbidity, or recurrence rate.

## 1. Introduction

The lifetime risk of developing ovarian cancer is low, at around 2% [1]. However, it remains the leading cause of death among gynecological cancers [2]. Even though ovarian malignant tumors are aggressive, they do not have any specific symptoms or signs in the early stages and can be overlooked [3]. So, the lack of awareness and the lack of prominent manifestations can lead to a delayed diagnosis at higher stages of the disease [4]. Only 20–25% of ovarian cancer patients are diagnosed in the early stages [5], while the majority are detected at an advanced stage [6]. This has a negative impact on the prognosis of the patients [6]. On the other hand, benign ovarian tumors comprise the majority of adnexal masses, especially in pre-menopausal women [4]. They also lack typical symptoms or signs, and a differential diagnosis from early-stage ovarian cancer or borderline tumors is sometimes difficult, occurring in approximately 10% of all cases [7]. Physicians should always keep in mind the common risk factors of ovarian cancer, such as positive family genetic history, lifestyle, fertility and breastfeeding, body mass index, gynecological-related diseases, and hormone replacement therapy, when investigating an adnexal mass [8,9].

The key to adnexal mass treatment is to avoid over- or undertreatment of the patients. Missing an ovarian cancer diagnosis (a false negative result) can lead to disease dissemination and a definitive surgery delay, due to a 4–6-week follow-up period with no surgical intervention or to a more conservative surgical technique. On the contrary, a misdiagnosis of ovarian cancer (a false positive result) can lead to unnecessary radical surgical interventions and increased patient anxiety. The goal is to optimize patient triage, so as not to operate on all adnexal masses, but to refer all suspicious ones to gynecological oncology centers for further treatment.

Many tools have been proposed for the differential diagnosis of adnexal masses, such as radiological and laboratory tests. CA-125 has been the most promising biomarker for ovarian cancer screening. However, its value in the early detection of the disease is limited because of its low sensitivity and the fact that it can be increased in benign cases (such as endometriosis) [10]. Furthermore, another promising biomarker is Human epididymis protein 4 (HE4), which has a role in the diagnosis, prognosis, and recurrence of ovarian cancer [11]. The combination of the above-mentioned biomarkers shows the highest efficiency, since CA-125 can correct the variations of HE4 that arise from smoking or contraception [12]. In addition, the Risk of Ovarian Malignancy Algorithm (ROMA) incorporates both CA-125 and HE4 values with the menopausal status of the women with adnexal masses. Specific cut-off points for pre- and post-menopausal women have been defined, leading to 75% specificity and 84% sensitivity [13]. On the other hand, magnetic resonance imaging (MRI) has been found to be superior to computed tomography (CT) for the visualization of the female genital tract [14]. Nevertheless, the method of choice for the assessment of adnexal masses is the transvaginal color Doppler ultrasound [15]. It has a high sensitivity, specificity, and diagnostic accuracy comparable to MRI, while being a cheap, quick, non-invasive, and easily reproducible technique [16]. The Assessment of Different Neoplasias in the adnexa (ADNEX) risk prediction model, which is based on three clinical and six ultrasound predictor variables, is proposed as the optimal tool for triaging all suspicious adnexal masses [17,18].

Furthermore, cancer waiting lists are prolonged and have become a serious healthcare issue in many countries [19]. Clinicians are forced to delay surgery in presumed benign adnexal masses with a low probability of malignancy. However, studies from other types of cancer, such as breast, colorectal, bladder, and melanoma, show a worse prognosis when surgery is delayed [20]. As a result, the combination of a delayed diagnosis and a longer waiting time for surgery may lead to a worse disease prognosis when the final histopathological diagnosis is ovarian cancer. The aim of this study is to evaluate the impact of surgery delays on survival rates and the oncological outcomes of early-stage [International Federation of Gynecology and Obstetrics (FIGO) stages I and II] ovarian cancer patients.

## 2. Materials and Methods

### 2.1. Study Characteristics

We retrospectively reviewed the medical records of all women that were examined in the out-patient clinic for adnexal masses; we chose those with suspicious masses that were finally treated in the 1st Department of Obstetrics and Gynecology and had a final diagnosis of early-stage (FIGO stage I and II) ovarian cancer. The time period studied was from January 2012 to December 2019. Wait times were defined as the time interval from the day of first examination in the outpatient clinic to the day of surgery. Two groups of patients were formed, with a cut-off point of 5 weeks. Demographic data, oncological characteristics, and follow-up information were harvested. The primary outcomes of our study were the postoperative complication and survival rates. A total of 336 consecutive patients were initially examined for an adnexal mass during this time interval. Approval was obtained from the Review Board of the hospital.

### 2.2. Patients

The inclusion criteria were as follows:

- Suspicious adnexal mass during differential diagnosis.
- Final histological confirmation of epithelial ovarian cancer.
- FIGO stage I or II.
- Surgical treatment at the 1st Department of Obstetrics and Gynecology.

The exclusion criteria were as follows:

- Presence of a synchronous neoplasm.
- Recurrent ovarian cancer.
- Missing important registry data.

**Figure 1:**
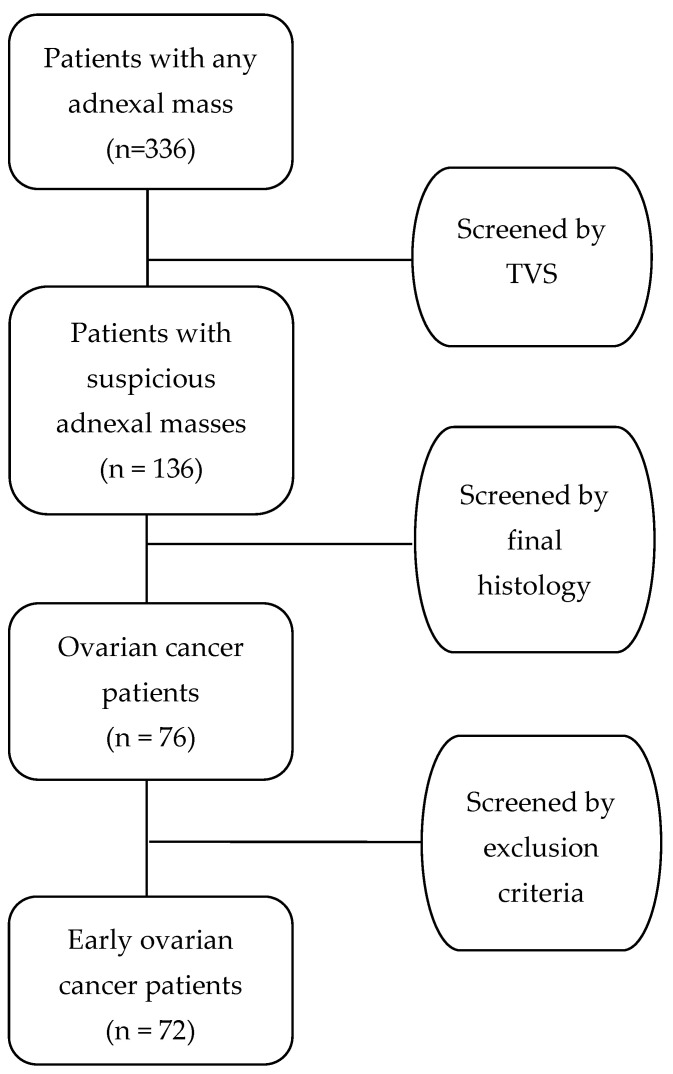
Patient selection flowchart.

As a result of the above-mentioned criteria, 200 women were excluded due to differential diagnosis of a probably benign adnexal mass. From the remaining 136 women, 60 were further dropped when the final histology revealed no evidence of malignancy. Furthermore, 2 out of the 76 women with a final diagnosis of an early-stage ovarian cancer were excluded due to recurrence, and another 2 women were excluded because they were missing important registry data. There was only one case of synchronous neoplasm (endometrial cancer), and that case was also excluded from the final analysis. Hence, 72 women with FIGO stage I and II ovarian cancer were finally identified as eligible for further analysis, with no duplicate data or important missing values. The flowchart of patient selection is analyzed in Figure 1.

**Figure 2:**
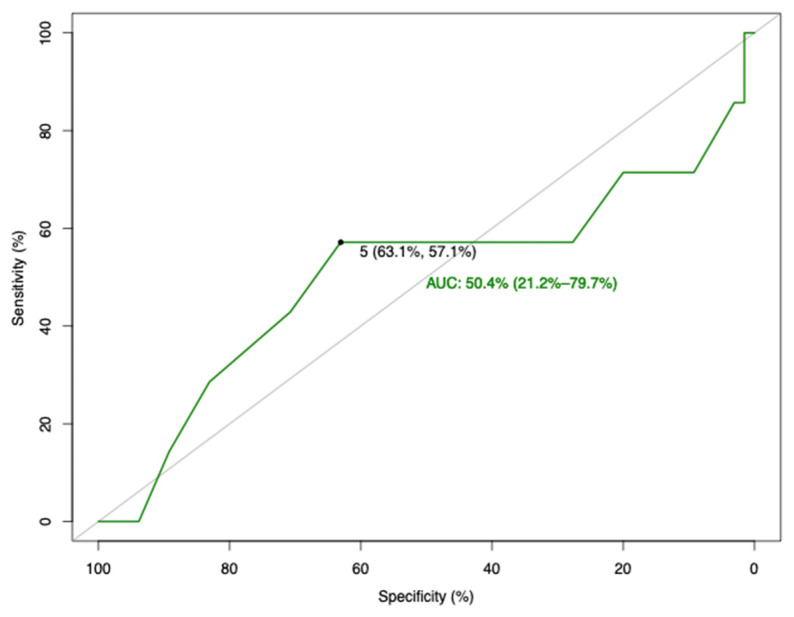
Surgery delay ROC curve (weeks).

The main reason for surgery delay in our study was the “wait and see” time period proposed for adnexal masses, which can range from 4 to 8 weeks. The cut-off point of 5 weeks in surgery delay was established using the receiver operating characteristics (ROC) curve analysis and the Youden index (J), based on disease recurrence (specificity: 63.1%, sensitivity: 57.1%, and AUC: 50.4%). The relevant data are presented in Figure 2. Patients were divided into 2 groups: Group A, which underwent surgery ≤ 5 weeks, and Group B, which underwent surgery > 5 weeks. The majority of the patients in Group B were operated on within 8 weeks, but some extreme cases of surgery delay were documented, mainly due to anesthesiologist shortages and severe patient comorbidities. All patients were treated with laparotomy, due to the characterization of a suspicious adnexal mass. Patients in both groups underwent total hysterectomy and bilateral salpingo-oophecetomy with infracolic omentectomy, pelvic and paraaortic lymphadenectomy, and peritoneal biopsies from the vesicouterine pouch, the paracolic gutters, and the hemidiaphragms. No residual disease was present in any of the cases that were included in the study. Last but not least, all patients after surgery were discussed in the multi-disciplinary team (MDT) meeting, and adjuvant systemic therapy was administrated nearly to all patients (n = 70, 97%), according to the European Society of Gynecological Oncology (ESGO) guidelines, after an approximate period of 30 days from surgery.

### 2.3. Data Collection

Patients’ medical records were reviewed in a time period of one month. All data were collected from an online registry. Data collection was performed by two investigators on different dates. So, to avoid inconsistencies, a uniform data collection sheet (excel file) was used during the retrospective mining of the patients’ medical records. The data sheet included the following information:

- Patient identifiers:
  ○ Name;
  ○ Hospital identification number.
- Patient’s age.
- Body Mass Index (BMI).
- Charlson Comorbidity Index (CCI).
- FIGO stage.
- Tumor marker CA-125 preoperative value.
- Histopathology.
- Intraoperative blood loss.
- Surgery duration.
- Intensive Care Unit (ICU) admission.
- Clavien–Dindo classification for post-operative complications.
- Hospital stay.
- Time-related data:
  ○ Date of first examination;
  ○ Date of surgery;
  ○ Date of recurrence;
  ○ Date of last follow-up or death.

### 2.4. Statistical Analysis

In the statistical analysis, the demographic data of the patients included in the study were calculated. For descriptive statistics of qualitative variables, the frequency distribution procedure was run with calculation of the number of cases and percentages. A test of normality was conducted using the Shapiro–Wilk test. On the other hand, for descriptive statistics of quantitative variables, the mean, median, range, and standard deviation were used to describe central tendency and dispersion. A receiver operating characteristic (ROC) curve analysis and the Youden index (J) [21,22] for disease recurrence were performed to identify the most appropriate cut-off value for surgery delay. The sensitivity, specificity, and area under the curve (AUC) with 95% confidence intervals (CI) were calculated. Disease-free (DFS) and overall survival (OS) analyses were measured with the Kaplan–Meier curves and group comparison was performed using the Cox regression test. Disease-free survival was defined as the time interval between the date of surgery and the date of first recurrence, while overall survival was defined as the time interval from the date of surgery to the date of death or last follow-up. *p*-value was set at a 5% significance level. We analyzed data using R statistical software (R Project for Statistical Computing), version 4.3.0, using the package called pROC [23].

## 3. Results

This retrospective cohort study included 76 women who underwent surgical treatment for early-stage ovarian cancer from 2012 to 2019, in the Gynecological–Oncology Unit, 1st Department of Obstetrics and Gynecology, Aristotle University of Thessaloniki, “Papageorgiou” General Hospital. After a thorough screening, 72 patients met the inclusion criteria for further analysis in this study.

Based on the 5-week cutoff point, patients were divided into two groups: 32 patients underwent surgery ≤ 5 weeks (Group A), and 40 patients > 5 weeks (Group B). Patient characteristics are designated in Table 1. Both groups of patients were approximately similar concerning the total number of women included in the study, with no significant difference in age, BMI, or comorbidities. The mean age of the women at the time of diagnosis was 51.6 years old for Group A and 53.9 for Group B, respectively. The majority of the patients were overweight, with an approximate median BMI at 26.9 kg/m^2^ and with mild comorbidities. On the other hand, a significant difference was observed in histology, even though most patients presented with serous or endometrioid ovarian cancer tumors, with higher rates of other histological subtypes in Group B compared to Group A (0% vs. 30%). Furthermore, even though the majority of the patients presented with FIGO stage I disease, there was significant difference between the two groups. This occurred because all six cases of patients with FIGO stage II disease underwent surgery > 5 weeks (Group B). However, no significant difference was detected concerning pre-operative CA-125 values (80 U/mL vs. 35.6 U/mL), intraoperative blood loss (300 cc vs. 250 cc), surgery duration (180 min vs. 150 min), postoperative complications measured with Clavien–Dindo classification (15 vs. 12.2) and hospital stay (7 days vs. 6 days). None of the patients required ICU admission in both groups.

**Figure 3:**
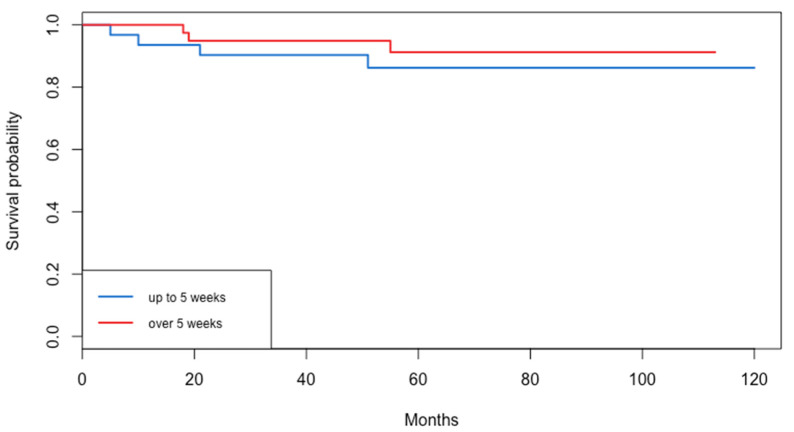
Disease-free survival.

**Figure 4:**
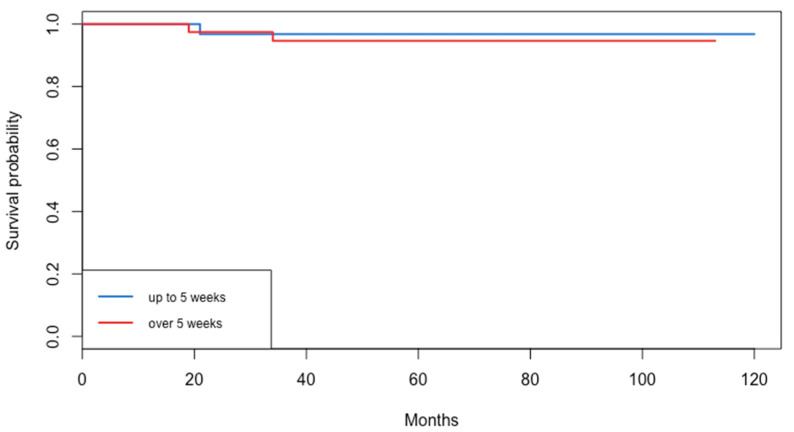
Overall survival.

The median follow-up time of the patients that were included in the cohort was 70.5 months with an IQR of 43.8–82.3. Concerning survival rates, no significant difference was observed in disease-free survival (*p* = 0.48) between the two groups. In total, seven patients suffered from a recurrence (four in Group A and three in Group B). Similarly, no significant difference was observed in overall survival (*p* = 0.703) between Group A and B. The median DFS and OS was >120 months for both groups. Survival data are presented in Figure 3 and Figure 4 for DFS and OS, respectively.

## 4. Discussion

The primary outcome of our study was to evaluate the impact of a 5-week surgery delay on the survival rates (DFS and OS) of early-stage (FIGO stage I and II) ovarian cancer. Secondary outcomes were considered the impact of surgery delay in the other oncological outcomes, such as intraoperative blood loss, surgery duration, post-operative complications, hospital stay and ICU admission rate. We designed a retrospective study, and all consecutive patients with early-stage ovarian cancer from 2012 to 2019 were meticulously triaged for the inclusion and exclusion criteria. Finally, 72 patients were available for further analysis. Based on surgery delay, the patients were divided into two groups with the cut-off point at 5 weeks. The specific cut-off point for surgery delay was calculated with ROC curve analysis, based on disease recurrence, which was one of the primary objectives of the study. Group A (≤5 weeks) included 32 patients and Group B (>5 weeks) included 40 patients. It is important to state that only seven (9.7%) patients suffered from a very long wait-time period and underwent surgery > 12 weeks.

Furthermore, the two groups of patients were similar and did not significantly differ in terms of age, BMI, or comorbidities. This strengthens the results of our study since possible selections bias are minimized. However, a significant difference was observed in FIGO stage and histology, even though the majority of the patients in both groups (n = 66, 91.7%) had FIGO stage I disease and most of them had either serous or endometrioid histological subtypes. These occurred because all cases (n = 6, 8.3%) of FIGO stage II disease and all cases other histological subtypes (n = 6, 8.3%) underwent surgery > 5 weeks and were included in Group B. On the other hand, no significant difference was found in the other secondary outcomes. Pre-operative CA-125 values, intraoperative blood loss, surgery duration, post-operative complications and hospital stay were similar among the two groups, while no patient out of the whole cohort required ICU admission. Similarly, concerning the survival rates, which was the primary outcome of our study, no significant differences were observed between the two groups. Disease-free survival and overall survival were not affected by surgery delay, and median DFS and OS were >120 months in both Group A and B. It is important to state that the surveillance period of the patients in the cohort was adequate to proceed with the survival analysis and obtain robust data, since the median follow-up was 70.5 months.

Reviewing the literature on the topic of surgery delay in ovarian cancer patients and its impact on the oncological outcomes, only a handful of studies are relevant. The number of studies decreased even further for the topic of early-stage ovarian cancer patients and the impact of surgery delay. In addition, the data presented in the literature are contradictory, because results vary from negative to positive and no impact on the patient’s survival rates as a result of surgery delay. The included studies from the literature are summarized in Table 2.

The most recent study is a systematic review [24] of the literature investigating the association of time to diagnosis and treatment for ovarian cancer patients. The authors conclude that variant time interval definitions exist in the literature, making it difficult to extract robust data and further analyze them. However, out of the nineteen studies that examined the impact of delay on survival rates, no association was found.

Furthermore, a retrospective analysis of ovarian cancer patients from the Surveillance, Epidemiology, and End Results (SEER) database (2010–2015) which included approximately 15,000 patients, around 50% had FIGO stage I and II disease [25]. They divided the patients into three groups, based on the treatment start time: <1 month, 1–2 months, and >3 months. Concerning FIGO stages, patients were equally distributed in the three groups, and the ones with advanced-stage disease were more likely to experience surgery delays. When subgroup analysis was performed on patients with early-stage ovarian cancer, those experiencing surgery delay had a higher risk of mortality. These results differ from those of our study, since we did not find any association between reduced overall survival and prolonged waiting time to surgery. This could be explained by the longer follow-up period of the other study and that the endpoint of the study was the time to treatment and not specifically surgery, which is also stated as a limitation by the authors. Moreover, a retrospective study that looked specifically at the impact of surgery delay in stage I ovarian cancer patients was presented as poster in 2021 [26]. The population was around 10,000 women with stage I ovarian cancer from the National Cancer Database (2004–2015) and had an even longer follow-up period than the abovementioned study. Patients were divided into two groups: surgery < 14 days, and surgery between 2 and 4 months. The authors found that surgery delay was not significantly associated with different overall survival, which is in accordance with our results.

In contrary, a retrospective study from the SEER database (1992–2015) [27] showed a waiting time paradox, meaning that longer time to treatment was associated with improved survival. However, this study included only FIGO stage II–IV patients. The authors conclude that referral to a gynecologic oncologist to guide appropriate treatment is more important than starting treatment as quickly as possible. Additionally, two studies [28,29] investigating the impact of the COVID-19 pandemic surgery delay on the patients’ outcomes found a reduced quality of life and a fewer diagnosis of stage I disease, but no data about survival. A nationwide study from Denmark [30] investigated the impact of surgery delay on the quality of life in both endometrial and ovarian cancer patients and found that some quality-of-life measures are affected by the total delay that the patients experienced. Last but not least, a systematic review of the literature in 2015 [20] about the impact of delay in diagnosis and treatment in all types of cancers showed that a shorter time to diagnosis leads to more favorable outcomes. Nevertheless, no robust data were presented about ovarian cancer patients and prolonged waiting times for diagnosis or treatment.

The present study took place in an academic, tertiary ESGO training center that was certified for advanced ovarian cancer surgery and gynecological oncology. All the required parameters were harvested from an online registry, leading to a very small percentage of important data that were missing. Furthermore, our study is the only cohort one with patients from a single center and not from a national database, where many valuable parameters are not available. On the other hand, the main limitation of our study was the small patient sample included in the final analysis and the fact that this was not a prospective study.

The results of our study add to the limited and contradictory literature about the impact of surgery delay in early-stage ovarian cancer and, in general, suspicious adnexal masses. The key suggestion to physicians examining patients with suspicious adnexal masses should be to refer them to gynecological oncology centers, and not to worry about interval referral time delays.

## 5. Conclusions

Differential diagnosis of adnexal masses should be made carefully. All available tools should be used to identify suspicious masses for malignancy, in order to avoid a prolonged “wait and see” period. However, even if the final histology reveals a malignancy, the data are reassuring. Prolonged surgery delay in early-stage ovarian cancer patients was not associated with reduced disease-free or overall survival. Patients with suspicious adnexal masses should be referred to gynecological oncology center, where meticulous TVS should be performed by an expert.

## Figures and Tables

**Table 1:** Patient characteristics.

Characteristics		Group A ≤ 5 Weeks n (%): 32 (44.4)	Group B > 5 Weeks n (%): 40 (55.6)	*p*-Value
Age (years) mean (SD)		51.6 (13.4)	53.9 (14.4)	0.4911
BMI (kg/m^2^) median (IQR)		26.9 (24.1, 29.9)	26.8 (24, 28.9)	0.6894
CCI median (IQR)		0.5 (0, 2)	1 (0, 2.3)	0.6455
CA-125 (U/mL) median (IQR)		80 (24.4, 419.1)	35.6 (12.6, 163.7)	0.1174
Blood loss (cc) median (IQR)		300 (200, 525)	250 (150, 375)	0.1575
Surgery duration (min) median (IQR)		180 (120, 240)	150 (120, 240)	0.4517
Histology				**0.00312**
	serous	20 (62.5%)	18 (45%)	
	endometrioid	12 (37.5%)	10 (25%)	
	other	0 (0%)	12 (30%)	
FIGO Stage				**0.03037**
	I	32 (100%)	34 (85%)	
	II	0 (0%)	6 (15%)	
Clavien–Dindo classification median (IQR)		15 (0, 16.5)	12.2 (0, 20.9)	0.7301
Hospital stay (days) median (IQR)		7 (6, 7.3)	6 (6, 7.3)	0.7939

**Table 2:** Relevant studies in the literature.

Studies	Year	Type	Survival Rates
Bergin et al. [24]	2024	Systematic review	No association
Zhao et al. [25]	2024	Retrospective	Negative association
Darling et al. [26]	2021	Retrospective	No association
Huepenbecker et al. [27]	2022	Retrospective	Positive association
Moterani et al. [28]	2022	Retrospective	No data
Frey et al. [29]	2020	Retrospective	No data
Robinson et al. [30]	2012	Retrospective	No data
Neal et al. [20]	2015	Systematic review	Negative association

## Data Availability

In accordance with the journal’s guidelines, the data presented in this study are available on request from the corresponding author for the reproducibility of this study if requested.
